# Supplementary material for: Chalcogen Bond Involving Zinc(II)/Cadmium(II) Carbonate and Its Enhancement by Spodium Bond
Source: Molecules. 2021 Oct 26;26(21):6443. doi: 10.3390/molecules26216443 (PMC8588527; doi:10.3390/molecules26216443)
Supplement: Supplementary file 1 [file molecules-26-06443-s001.zip › molecules-1414571-supplementary.pdf]

Table S1 Angles (α, deg) and binding distances (R, Å) in the ternary complexes as well as their difference (Δ) relative to the binary analogues

	R_{SpB}	R_{ChB}	R_{HB}	α_1	α_2	$\Delta\alpha_1$	$\Delta\alpha_2$
T-1	1.907	2.418	2.343	169.7	124.3	0.6	-0.7
T-2	2.118	2.379	2.312	169.7	124.0	0.4	-0.5
T-3	1.908	2.413	2.341	169.6	124.3	0.5	-0.7
T-4	2.116	2.369	2.301	169.7	123.8	0.4	-0.7
T-5	1.942	2.410	2.336	169.5	124.4	0.4	-0.6
T-6	2.143	2.371	2.302	169.6	124.1	0.3	-0.4
T-7	1.907	2.521	2.273	167.7	127.6	1.0	-1.2
T-8	2.119	2.468	2.235	168.0	127.0	0.9	-1.2
T-9	1.908	2.515	2.273	167.9	127.3	1.2	-1.5
T-10	2.117	2.453	2.231	168.2	126.5	1.1	-1.7
T-11	1.942	2.511	2.264	167.7	127.6	1.0	-1.2
T-12	2.147	2.456	2.233	168.1	126.8	1.0	-1.4
T-13	1.909	2.649	2.354	167.6	130.2	0.9	0.4
T-14	2.121	2.610	2.318	167.5	130.2	0.6	0.4
T-15	1.910	2.645	2.353	167.5	130.3	0.8	0.5
T-16	2.110	2.615	2.310	167.6	130.1	0.7	0.3
T-17	1.944	2.641	2.348	167.4	130.4	0.7	0.6
T-18	2.146	2.602	2.309	167.5	130.2	0.6	0.4
T-19	1.909	2.658	2.355	167.3	130.5	0.5	0.1
T-20	2.121	2.617	2.315	167.4	130.2	0.7	-0.3
T-21	1.910	2.654	2.353	167.4	130.4	0.6	0
T-22	2.110	2.623	2.308	167.5	130.1	0.8	-0.4
T-23	1.945	2.649	2.349	167.4	130.3	0.6	-0.1
T-24	2.149	2.607	2.303	167.3	130.4	0.6	-0.1
T-25	1.910	2.906	2.285	160.0	139.7	0.2	0.8
T-26	2.123	2.876	2.234	159.8	140.2	-0.2	1.3
T-27	1.911	2.900	2.285	160.0	139.7	0.2	0.8
T-28	2.112	2.875	2.238	160.2	139.6	0.2	0.7
T-29	1.946	2.903	2.270	159.8	140.1	0	1.2
T-30	2.149	2.873	2.221	159.6	140.6	-0.4	1.7
T-31	1.910	2.931	2.271	159.8	140.7	0	1.1
T-32	2.123	2.904	2.213	159.3	141.6	0.1	0.8
T-33	1.911	2.925	2.273	160.0	140.3	0.2	0.7
T-34	2.112	2.907	2.220	161.0	138.3	1.8	-2.5
T-35	1.946	2.929	2.255	159.5	141.2	-0.3	1.6
T-36	2.146	2.921	2.194	158.3	142.2	-0.9	1.4

Table S2 Electron densities (ρ, au) at the intermolecular BCPs in the ternary systems and their change (Δρ, au) relative to the binary analogues.

	ρ_{ChB}	ρ_{SpB}	$\Delta\rho_{ChB}$	$\Delta\rho_{SpB}$
T-1	0.0383	0.1042	0.0071	0.0018
T-2	0.0420	0.0855	0.0066	0.0056
T-3	0.0387	0.1127	0.0075	0.0017
T-4	0.0429	0.0931	0.0075	0.0034
T-5	0.0391	0.1055	0.0079	0.0026
T-6	0.0428	0.0891	0.0074	0.0059
T-7	0.0319	0.1042	0.0071	0.0018
T-8	0.0360	0.0854	0.0073	0.0055
T-9	0.0323	0.1126	0.0075	0.0016
T-10	0.0372	0.0930	0.0085	0.0033
T-11	0.0327	0.1048	0.0079	0.0019
T-12	0.0370	0.0878	0.0083	0.0046
T-13	0.0238	0.1035	0.0042	0.0011
T-14	0.0260	0.0848	0.0040	0.0049
T-15	0.0239	0.1121	0.0043	0.0011
T-16	0.0256	0.0947	0.0036	0.0050
T-17	0.0242	0.1041	0.0046	0.0012
T-18	0.0265	0.0882	0.0045	0.0050
T-19	0.0234	0.1035	0.0042	0.0011
T-20	0.0257	0.0848	0.0041	0.0049
T-21	0.0236	0.1121	0.0044	0.0011
T-22	0.0253	0.0947	0.0037	0.0050
T-23	0.0239	0.1041	0.0047	0.0012
T-24	0.0263	0.0877	0.0047	0.0045
T-25	0.0145	0.1031	0.0019	0.0007
T-26	0.0156	0.0844	0.0016	0.0045
T-27	0.0147	0.1117	0.0021	0.0007
T-28	0.0155	0.0943	0.0015	0.0046
T-29	0.0147	0.1043	0.0021	0.0014
T-30	0.0157	0.0877	0.0017	0.0045
T-31	0.0140	0.1032	0.0019	0.0008
T-32	0.0149	0.0844	0.0016	0.0045
T-33	0.0141	0.1118	0.0020	0.0008
T-34	0.0149	0.0943	0.0016	0.0046
T-35	0.0141	0.1037	0.0020	0.0008
T-36	0.0143	0.0882	0.0010	0.0050

Table S3 Laplacians ($\nabla^2\rho$) and energy densities (H) at the intermolecular Se···O and M···N BCPs in the ternary complexes, all in au.

	Se···O		M···N	
	$\nabla^2\rho$	H	$\nabla^2\rho$	H
T-1	0.1188	-0.0028	0.4604	-0.0390
T-2	0.1242	-0.0043	0.3599	-0.0239
T-3	0.1199	-0.0029	0.4174	-0.0473
T-4	0.1255	-0.0046	0.3370	-0.0300
T-5	0.1198	-0.0031	0.3772	-0.0426
T-6	0.1249	-0.0046	0.3053	-0.0281
T-7	0.1025	-0.0006	0.4607	-0.0389
T-8	0.1100	-0.0018	0.3598	-0.0239
T-9	0.1036	-0.0007	0.4172	-0.0473
T-10	0.1123	-0.0022	0.3369	-0.0299
T-11	0.1040	-0.0008	0.3801	-0.0420
T-12	0.1117	-0.0022	0.3052	-0.0274
T-13	0.0846	0.0011	0.4591	-0.0385
T-14	0.0902	0.0007	0.3585	-0.0235
T-15	0.0854	0.0010	0.4149	-0.0469
T-16	0.0896	0.0008	0.3299	-0.0313
T-17	0.0858	0.0010	0.3791	-0.0415
T-18	0.0912	0.0006	0.3040	-0.0276
T-19	0.0831	0.0011	0.4590	-0.0385
T-20	0.0889	-0.0007	0.3585	-0.0235
T-21	0.0838	0.0011	0.4148	-0.0469
T-22	0.0882	0.0008	0.3300	-0.0313
T-23	0.0844	0.0010	0.3790	-0.0415
T-24	0.0903	0.0006	0.3016	-0.0273
T-25	0.0548	0.0017	0.4585	-0.0382
T-26	0.0581	0.0017	0.3577	-0.0232
T-27	0.0556	0.0017	0.4137	-0.0467
T-28	0.0582	0.0017	0.3289	-0.0311
T-29	0.0552	0.0017	0.3757	-0.0417
T-30	0.0585	0.0017	0.3029	-0.0273
T-31	0.0525	0.0017	0.4586	-0.0382
T-32	0.0554	0.0017	0.3577	-0.0232
T-33	0.0531	0.0017	0.4138	-0.0467
T-34	0.0557	0.0017	0.3288	-0.0311
T-35	0.0528	0.0017	0.3786	-0.0412
T-36	0.0539	0.0017	0.3063	-0.0274

Table S4 The most negative MEP ($V_{S,min}$) on the O atom of C=O bond in the spodium-bonded binary complex and the most positive MEP ($V_{S,max}$) on the M atom in the chalcogen-bonded binary complex as well as their difference (ΔV) relative to the corresponding monomers, all in kcal/mol.

dyads	$V_{S,max}$	$\Delta V_{S,max}$	dyads	$V_{S,min}$	$\Delta V_{S,min}$
ChB-1	176.10	13.69	HCN-$ZnCO_3$	-73.14	-13.96
ChB-2	156.83	12.51	HCN-$CdCO_3$	-78.38	-11.70
ChB-3	174.81	12.40	H_2CHN-$ZnCO_3$	-72.51	-13.33
ChB-4	155.92	11.60	H_2CHN-$CdCO_3$	-77.61	-10.93
ChB-5	170.25	7.84	H_3N-$ZnCO_3$	-72.26	-13.08
ChB-6	151.59	7.27	H_3N-$CdCO_3$	-78.14	-11.46
ChB-7	170.34	7.93			
ChB-8	151.60	7.28			
ChB-9	166.76	4.35			
ChB-10	148.07	3.75			
ChB-11	166.71	4.30			
ChB-12	148.09	3.77			

Table S5 Charge transfer (CT, e) of chalcogen and spodium bonds in the ternary complexes and its change (ΔCT, e) relative to the binary analogues

	CT_{ChB}	CT_{SpB}	ΔCT_{ChB}	ΔCT_{SpB}
T-1	0.0571	0.1230	0.0181	0.0025
T-2	0.0671	0.0822	0.0178	0.0045
T-3	0.0569	0.1566	0.0179	0.0025
T-4	0.0689	0.1241	0.0196	0.0048
T-5	0.0587	0.1596	0.0197	0.0036
T-6	0.0691	0.1063	0.0198	0.0052
T-7	0.0486	0.1229	0.0180	0.0024
T-8	0.0593	0.0822	0.0189	0.0045
T-9	0.0486	0.1565	0.0180	0.0024
T-10	0.0618	0.1239	0.0214	0.0046
T-11	0.0503	0.1593	0.0197	0.0033
T-12	0.0618	0.1063	0.0214	0.0052
T-13	0.0274	0.1221	0.0094	0.0016
T-14	0.0325	0.0812	0.0093	0.0035
T-15	0.0270	0.1558	0.0090	0.0017
T-16	0.0306	0.1236	0.0074	0.0043
T-17	0.0281	0.1581	0.0101	0.0021
T-18	0.0336	0.1050	0.0104	0.0039
T-19	0.0279	0.1220	0.0093	0.0015
T-20	0.0332	0.0811	0.0095	0.0034
T-21	0.0276	0.1557	0.0090	0.0016
T-22	0.0314	0.1236	0.0077	0.0043
T-23	0.0288	0.1580	0.0102	0.0020
T-24	0.0345	0.1049	0.0108	0.0038
T-25	0.0139	0.1216	0.0048	0.0011
T-26	0.0165	0.0805	0.0045	0.0028
T-27	0.0134	0.1551	0.0043	0.0010
T-28	0.0154	0.1229	0.0034	0.0036
T-29	0.0143	0.1573	0.0052	0.0013
T-30	0.0172	0.1042	0.0052	0.0031
T-31	0.0138	0.1215	0.0046	0.0010
T-32	0.0164	0.0805	0.0046	0.0028
T-33	0.0134	0.1550	0.0042	0.0009
T-34	0.0153	0.1228	0.0035	0.0035
T-35	0.0142	0.1573	0.0050	0.0013
T-36	0.0161	0.1029	0.0043	0.0018

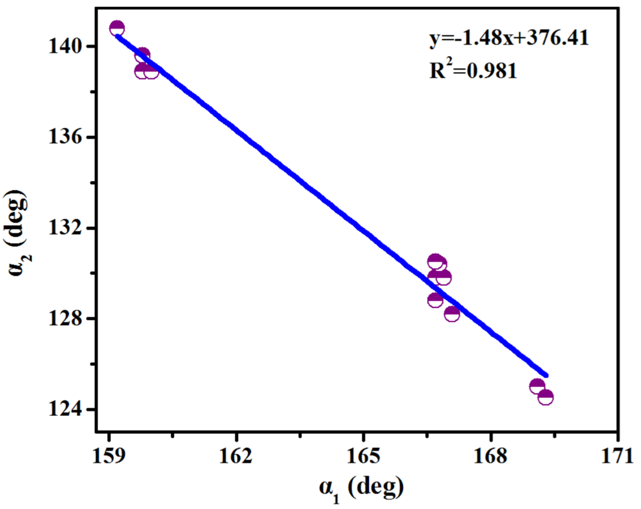

Figure S1 Relationship between both angles of Se-H···O (α_2) and X-Se···O (α_1)

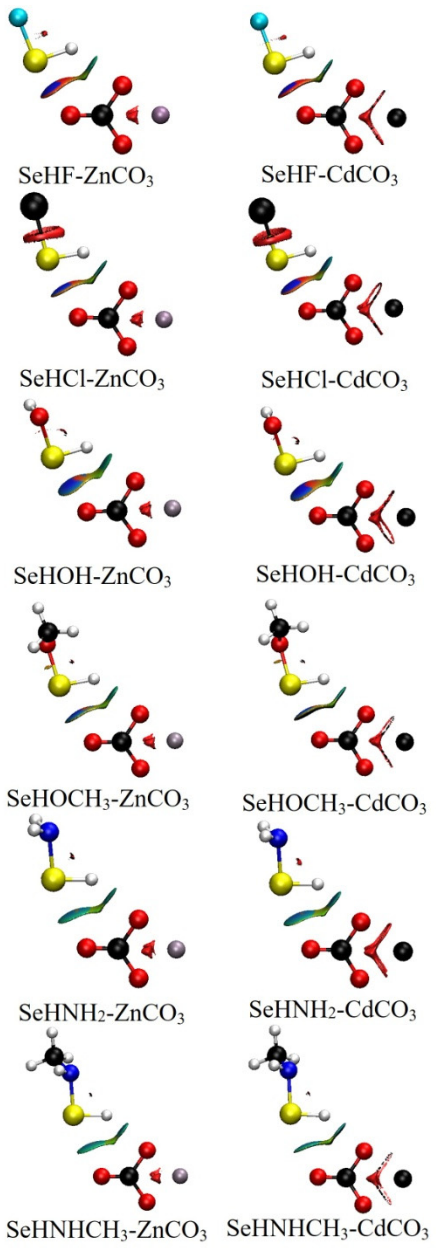

Figure S2 Non-covalent interaction (NCI) maps of the chalcogen-bonded dyads. Blue, green, and red areas correspond to strong attractive, weak attractive and strong repulsion interactions, respectively.

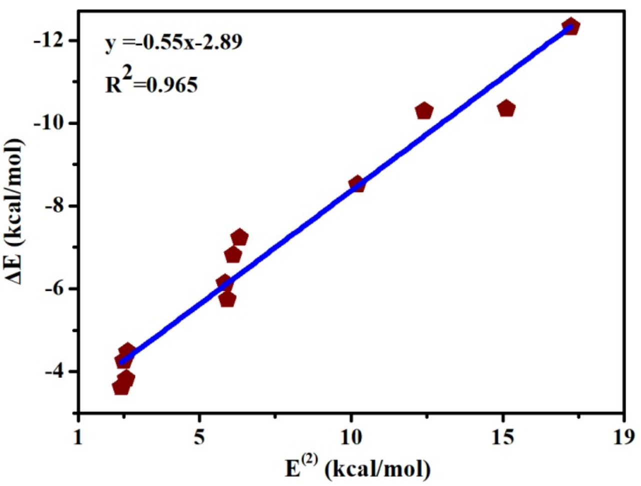

Figure S3 Interaction energy (ΔE) versus second-order perturbation energy ($E^{(2)}$) of $Lp_O \rightarrow \sigma^*_{Se\text{-}X}$ orbital interaction in the chalcogen-bonded dyads

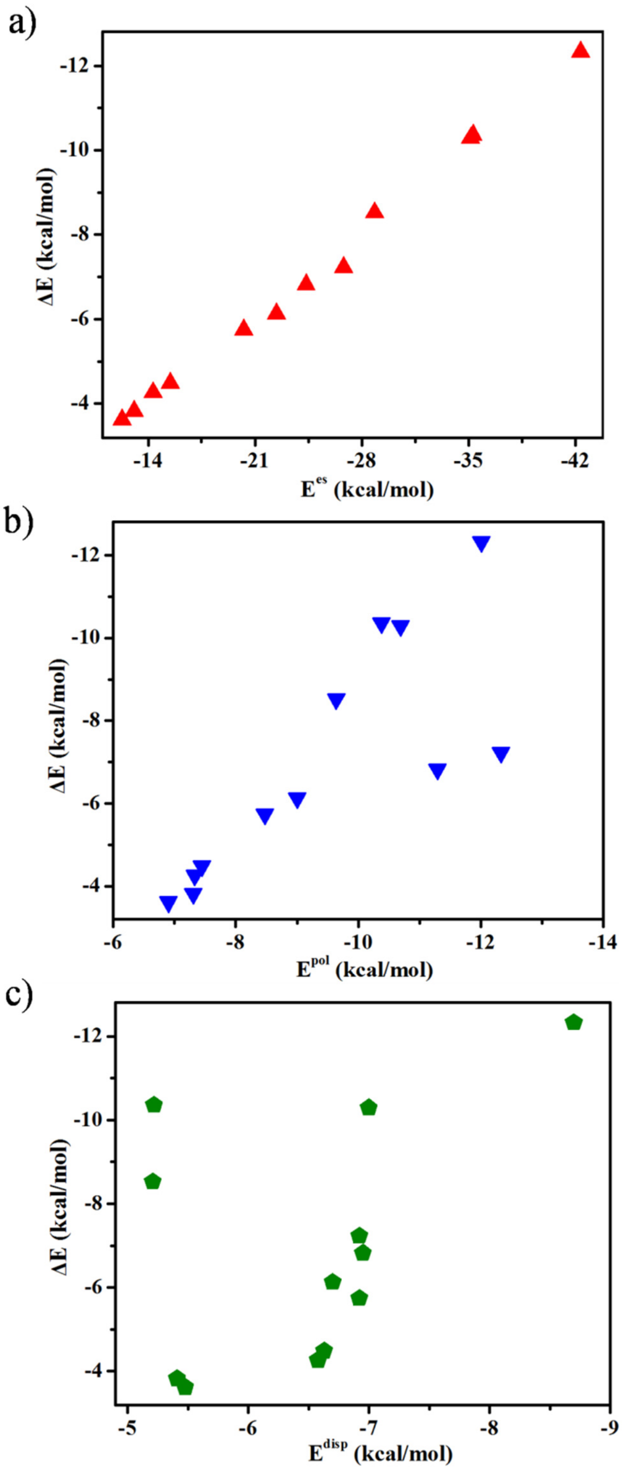

Figure S4 Interaction energy (ΔE) versus a) electrostatic energy (E^{es}), b) polarization energy (E^{pol}), and c) dispersion energy (E^{disp}) in the chalcogen-bonded dyads

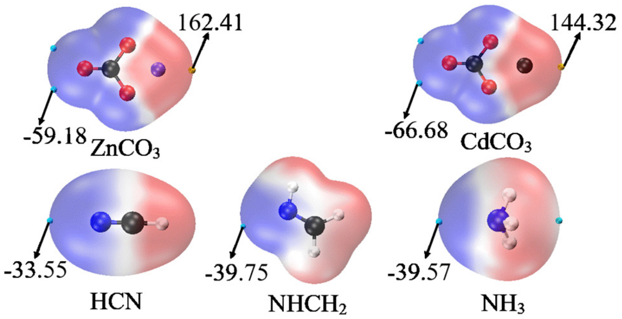

Figure S5 MEP maps on the 0.001 electrons Bohr^{-3} isodensity surface of monomers. Red and blue regions represent positive and negative MEPs, respectively. All are in kcal/mol.
